# Enhanced immune response outperform aggressive cancer biology and is associated with better survival in triple-negative breast cancer

**DOI:** 10.1038/s41523-022-00466-2

**Published:** 2022-08-09

**Authors:** Masanori Oshi, Ankit Patel, Rongrong Wu, Lan Le, Yoshihisa Tokumaru, Akimitsu Yamada, Li Yan, Ryusei Matsuyama, Takashi Ishikawa, Itaru Endo, Kazuaki Takabe

**Affiliations:** 1 Department of Surgical Oncology, Roswell Park Comprehensive Cancer Center, Buffalo, NY 14263 USA; 2 Department of Gastroenterological Surgery, Yokohama City University Graduate School of Medicine, Yokohama, 236-0004 Japan; 3 Department of Breast Surgery and Oncology, Tokyo Medical University, Tokyo, 160-8402 Japan; 4 Department of Surgical Oncology, Graduate School of Medicine, Gifu University, 1-1 Yanagido, Gifu, 501-1194 Japan; 5 Department of Biostatistics and Bioinformatics, Roswell Park Comprehensive Cancer Center, Buffalo, NY 14263 USA; 6 Division of Digestive and General Surgery, Niigata University Graduate School of Medical and Dental Sciences, Niigata, 951-8520 Japan; 7 Department of Breast Surgery, Fukushima Medical University School of Medicine, Fukushima, 960-1295 Japan; 8 Department of Surgery, Jacobs School of Medicine and Biomedical Sciences, State University of New York, Buffalo, NY 14263 USA

**Keywords:** Cell signalling, Tumour immunology, Breast cancer

## Abstract

Although the value of tumor-infiltrating lymphocytes is well known, the clinical relevance of an increased immune response, specifically in breast cancer, has not been investigated across large cohorts of patients using computational algorithms. Our hypothesis stated that an enhanced immune response is associated with an improvement in outcomes. To quantify the immune response, we utilized the allograft rejection score correlated with cytolytic activity and with all the other Hallmark immune-related gene sets. The score reflected the amount of infiltrating immune cells that correlated with the immune checkpoint molecule expressions, including CD4^+^ and CD8^+^ T cells, T helper type 1 (Th1) and type 2 (Th2) cells, M1 macrophages, B cells, and plasmacytoid dendritic cells (pDC). A high score was associated with high levels of intratumor heterogeneity, homologous recombination defects, mutation rate, histological grade, advanced stage, and lymph node metastasis. Breast malignancy with a high score enriched immune-related gene sets and pro-cancer-related gene sets, including epithelial–mesenchymal transition and KRAS pathway, in ER-positive/HER2-negative and triple-negative breast cancer (TNBC) groups. TNBC had the highest score compared to other subtypes, and was associated with better survival. In conclusion, we found that breast cancer with a high immune response is associated with aggressive cancer biology, but with better survival in TNBC.

## Introduction

Historically, cancer has been diagnosed by the combination of pathologic analyses and clinical parameters of cancer aggressiveness. With advances in molecular biology, genomic mutations have more recently become important in both the diagnosis and treatment of cancer. The measure of carcinogenesis—the process whereby normal cells transform into cancer cells via accumulation of DNA mutations—has become a foundational notion of precision medicine^1^. There are numerous mechanisms that cause genomic mutations, including homologous recombination defects (HRD), and cancers with a high mutational burden and greater intratumor heterogeneity have an increased risk of treatment-resistance. At the same time, a high tumor mutational burden has been suggested to increase neoantigen generation that can initiate an anti-cancer immune cell infiltration^2,3^. Indeed, we have previously reported that aggressive cancer biology and anti-cancer immunity is counterbalanced in breast cancers with high mutation rates^4^. Although tumor-infiltrating lymphocytes have been shown to associate with treatment response and prognosis^5,6^ pathologically by cell density and gene expression in some large studies of breast cancer^6,7^, no study has investigated the clinical relevance of an enhanced immune response using multiple computational algorithms on transcriptomes validated by multiple large breast cancer patient cohorts.

Immune cell infiltration in the tumor microenvironment (TME) strongly influences breast cancer biology and its treatment response^8^. Although previous studies have elegantly shown pathologically that existence of tumor-infiltrating lymphocytes (TIL) are known to predict patient survival in TNBC^9,10^, it remains unclear whether the number of immune cell infiltrations or the function of immune cells is associated with patient survival. Biomedical research is evolving rapidly to revolutionize the way molecular data is obtained and examined. Analyses of the gene expression profile of a bulk (whole) tumor utilizing computational algorithms is allowing us to grasp the immune condition in a human cancer TME, which is difficult, if not impossible, to completely reproduce through in vivo or in vitro experimental settings. For example, the cytolytic activity score (CYT), reported by Rooney et al.^11^, is a useful measure that estimates immune cell killing by analyzing the expressions of granzyme A (*GZMA*) and perforin (*PRF1*) genes in transcriptomes. We believe it is one of the most authenticated algorithms to estimate immune cell killing^12,13^, and we have previously confirmed its clinical relevance in colon cancer^14^ and liver cancer^15^. There are a number of computational algorithms that quantify the fraction of infiltrating immune cells in TME using tens to hundreds of cell marker gene expressions such as xCell^16^ and CIBERSORT^17^. Our group and the others have repeatedly shown that the competitive scoring of biological pathways using multiple genes can provide a more accurate understanding of cancer biology than any single gene expression analysis because multiple genes are often involved in cancer progression^18–21^. A score that utilizes multiple gene expression profiles from a single gene set reduces model complexity, takes the coordination of genes into account, and increases the explanatory power of prediction models^22–24^. To this end, our group has been utilizing the Gene Set Variation Analysis (GSVA) score of the Molecular Signatures Database (MSigDB) hallmark gene set collection to explore the biological activity in TME in the bulk tumors^25^. This method has allowed us to investigate the clinical relevance of multiple pathways from global transcriptomes such as E2F targets, G2M checkpoint, inflammatory response, angiogenesis, and interferon (IFN)-γ response pathway in breast cancer.

Here, we hypothesize that the Hallmark allograft rejection score, which strongly reflects anti-cancer immune activity and infiltration of immune cells, is associated with better patient outcomes. We identified that the immune response was strongly reflected by the Hallmark allograft rejection gene set among the immune-related GSVA scores in the MSigDB Hallmark gene sets collection. We investigated a total of 6245 breast cancer patients in experimental and validation cohorts to test our hypothesis.

## Results

### The allograft rejection score correlated strongly with cytolytic activity and the other immune-related gene sets, which suggests that it reflects anti-cancer immunity

**Fig. 1:**
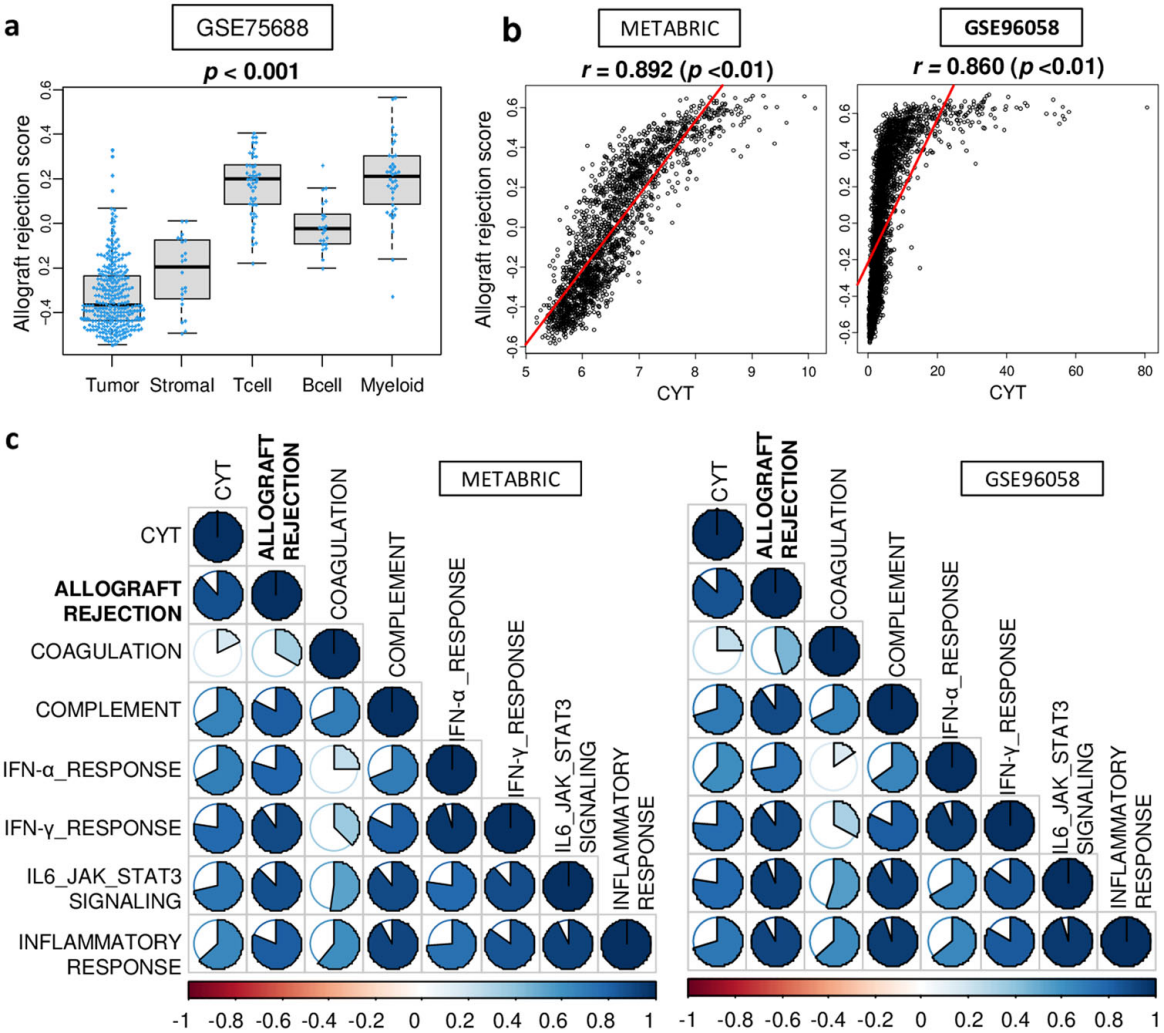
Allograft rejection score strongly reflected anti-cancer immunity in breast cancer. **a** Boxplots of the allograft rejection score by single cells of the tumor, stromal cells, T cells, B cells, and myeloid cells in the GSE75688 cohort. *p*-values were calculated by the Kruskal–Wallis test. Boxplots were of Tukey type, with boxes depicting median and inter-quartile range. **b** Scatter plots between Allograft rejection score and CYT score in both cohorts. Spearman rank correlation was used for the analysis. **c** Correlation plots between score level of CYT and 8 immune-related gene sets, including allograft rejection, coagulation, complement, interferon (IFN)-α, IFN-γ, IL6/JAK/STAT3/Signaling, and inflammatory response in the METABRIC and GSE96058 cohorts. The correlation value is indicated by color (blue for positive correlation and red for negative correlation), while the magnitude of the correlation is shown with circles.

The allograft rejection score (Supplementary Table 1) was defined by the Gene set variation analysis (GSVA) algorithm as one of the immune-related Hallmark gene sets in molecular signatures database (MSigDB)^26^, similar to how we defined the other scores in our previous publications^27–31^. To identify which cell types contribute to the allograft rejection score in the tumor microenvironment (TME), the score was measured in a single-cell sequence cohort (GSE75688) that has transcriptomes of tumor cells, stromal cells, T cells, B cells, and myeloid cells. A strong separation in the score was seen between the immune cells. T cells and myeloid cells had higher scores than tumor and stromal cells (Fig. 1a; *p* < 0.001). Next, we investigated how well the score reflected the immune response in the TME of breast cancer. We found that the score was strongly correlated with the cytolytic activity score (CYT), which reflects immune cell killing (Fig. 1b, Spearman rank test (*r*) = 0.892 and 0.860, respectively, both *p* < 0.01). The score was also correlated with the other immune-related gene sets scores consistently in both METABRIC and GSE96058 cohorts, including complement, interferon (IFN)-γ response, IFN-α response, IL6/JAK/STAT3 signaling, and inflammatory response, but not with coagulation (Fig. 1c). These findings suggest that the allograft rejection score strongly reflects anti-cancer immunity in TME.

### The allograft rejection score reflected the amount of infiltrating immune cells, specifically anti-cancer immune cells

**Fig. 2:**
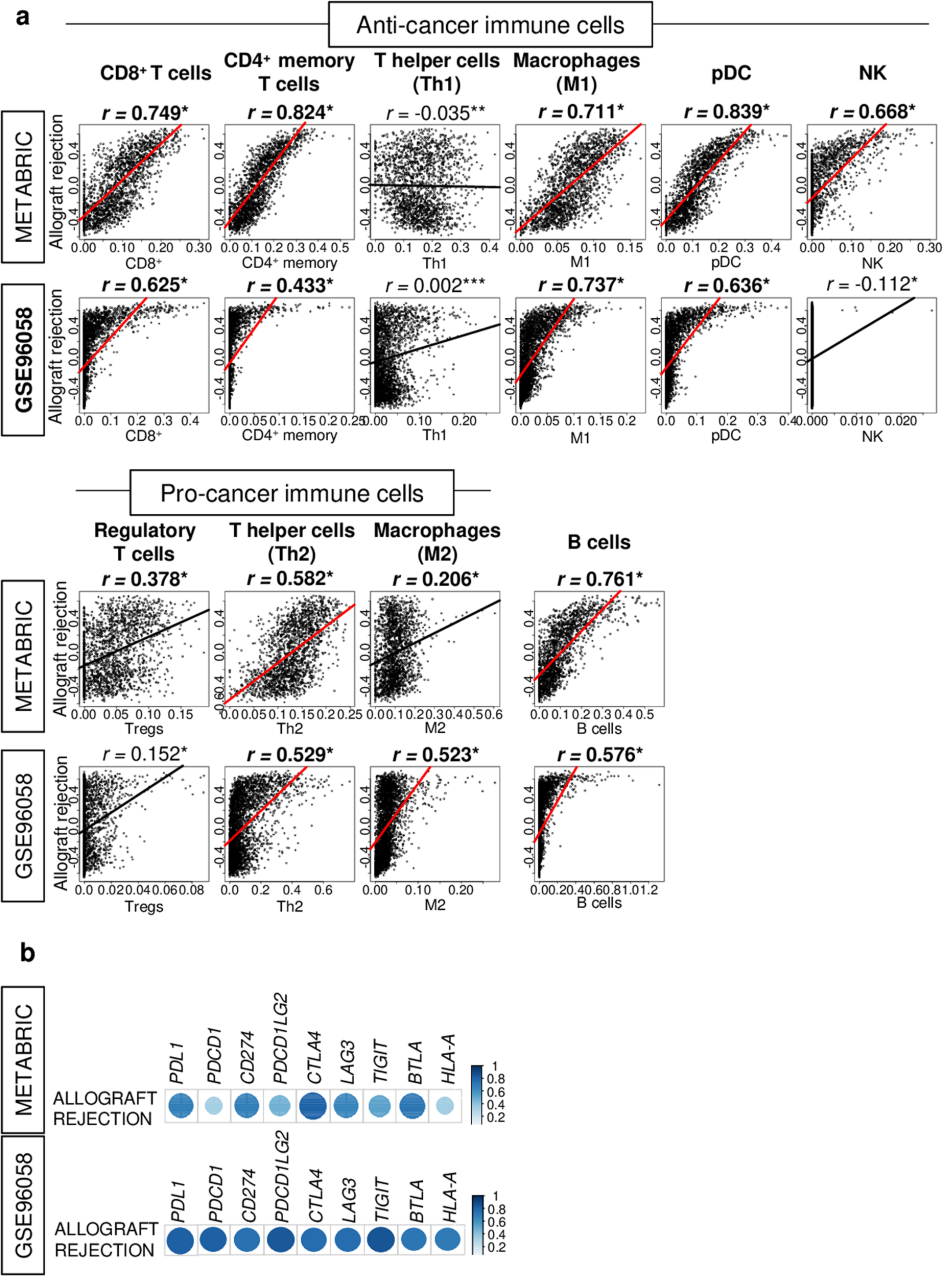
The allograft rejection score was strongly correlated with infiltration of immune cells. **a** Scatter plots between allograft rejection score and the fraction of anti-cancer immune cells; CD8^+^ T cells, CD4^+^ memory T cells, type1 T helper (Th1) cells, M1 macrophages, and pro-cancer immune cells; regulatory T cells (Tregs), type 2 T (Th2) cells, M2 macrophages, and B cells, calculated by xCell algorithm, in the METABRIC and GSE96058 cohorts. **b** Correlation plots between allograft rejection score and expression of immune checkpoint molecules, including *PDCD1/PD-1, CD274/PD-L1, PDCD1LG2/PD-L2, CTLA4, LAG3, TIGIT, BTLA,* and *HLA-A* in both cohorts. Spearman rank correlation was used for the analysis. *p < 0.01, **p = 0.13, ***p = 0.91.

Given that the allograft rejection score offers the strongest reflection of the anti-cancer immunity in the TME amongst all the Hallmark immune-related gene sets (Fig. 1), we decided to focus on the allograft rejection score in breast cancer. In addition to immune function, it was of interest to investigate whether the allograft rejection score is associated with immune cell infiltration in the TME. The xCell algorithm was used to estimate immune cell infiltration, as performed in previous publications^32–36^. We found that the score strongly correlated with the infiltration of anti-cancer immune cells in the METABRIC and GSE96058 cohorts, including CD8^+^ T cells, CD4^+^ memory T cells, M1 macrophages, and plasmacytoid dendritic cells (pDC) (Fig. 2a; all spearman’s rank correlation (*r*) > 0.700, all *p* < 0.01), but not with T helper type 1 (Th1) cells or natural killer (NK) cells. The score was correlated with T helper type 2 (Th2) cells (pro-cancer immune cells) and B cells, in both cohorts, but not with other pro-cancer immune cells (Fig. 2a). Furthermore, the score also correlated with the expression level of immune checkpoint molecules in both cohorts (Fig. 2b). These results suggest that the score is strongly correlated with infiltration of anti-cancer immune cells in breast cancer. Furthermore, we showed the association of other immune-related gene sets with infiltration fraction of immune cells in Supplementary Table 2. We found that allograft rejection score correlated strongly with not only cytolytic activity but also infiltration fraction of several immune cells compared to other immune-related gene sets.

### Breast cancer patients with a high allograft rejection score were associated with homologous recombination defect (HRD), high intratumor heterogeneity, and mutation rate

**Fig. 3:**
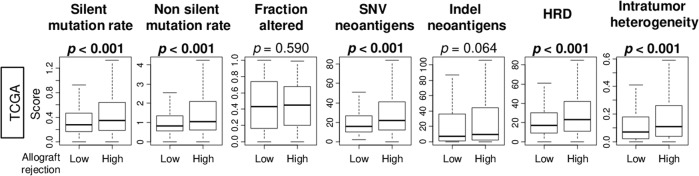
Breast cancer with a high allograft rejection score was significantly associated with high levels of the mutation-related score and intratumor heterogeneity in the TCGA cohort. Boxplots of the mutation-related scores; silent and non-silent mutation rate, fraction altered, single nucleotide variation (SNV) and indel neoantigens, homologous recombination defects (HRD), and intratumor heterogeneity by low and high allograft rejection score groups. The median value was used as a cut-off to divide two score groups. *p*-values were calculated by the Mann–Whitney U test. Boxplots were of Tukey type, with boxes depicting median and inter-quartile range.

It is well known that breast cancer with high tumor mutational burden generate neoantigens, which attract tumor-infiltrating immune cells into the TME. Our group have previously shown that mutation rate high breast cancer with aggressive cancer biology was counterbalanced by elevated immune cell infiltration^4^. To this end, it was of interest to investigate the relationship of the allograft rejection score with the mutation rate, intratumor heterogeneity, and DNA repair mechanisms such as HRD in breast cancer. We divided high and low allograft score groups by median within each cohorts. We found that breast cancer with a high score was significantly associated with both silent and non-silent mutation rates, single nucleotide variation (SNV) neoantigens, as well as HRD and intratumor heterogeneity in the TCGA cohort (Fig. 3). The details of the results are shown in Supplementary Table 3. This result is in agreement with our observation that high score breast cancer with increased immune cell infiltration and immune response is associated with high mutation rate, neoantigens, HRD and intratumoral heterogeneity, common in aggressive cancer.

### Breast cancer with a high allograft rejection score was significantly associated with advanced Nottingham histological grade, advanced stage, and lymph node metastasis

**Fig. 4:**
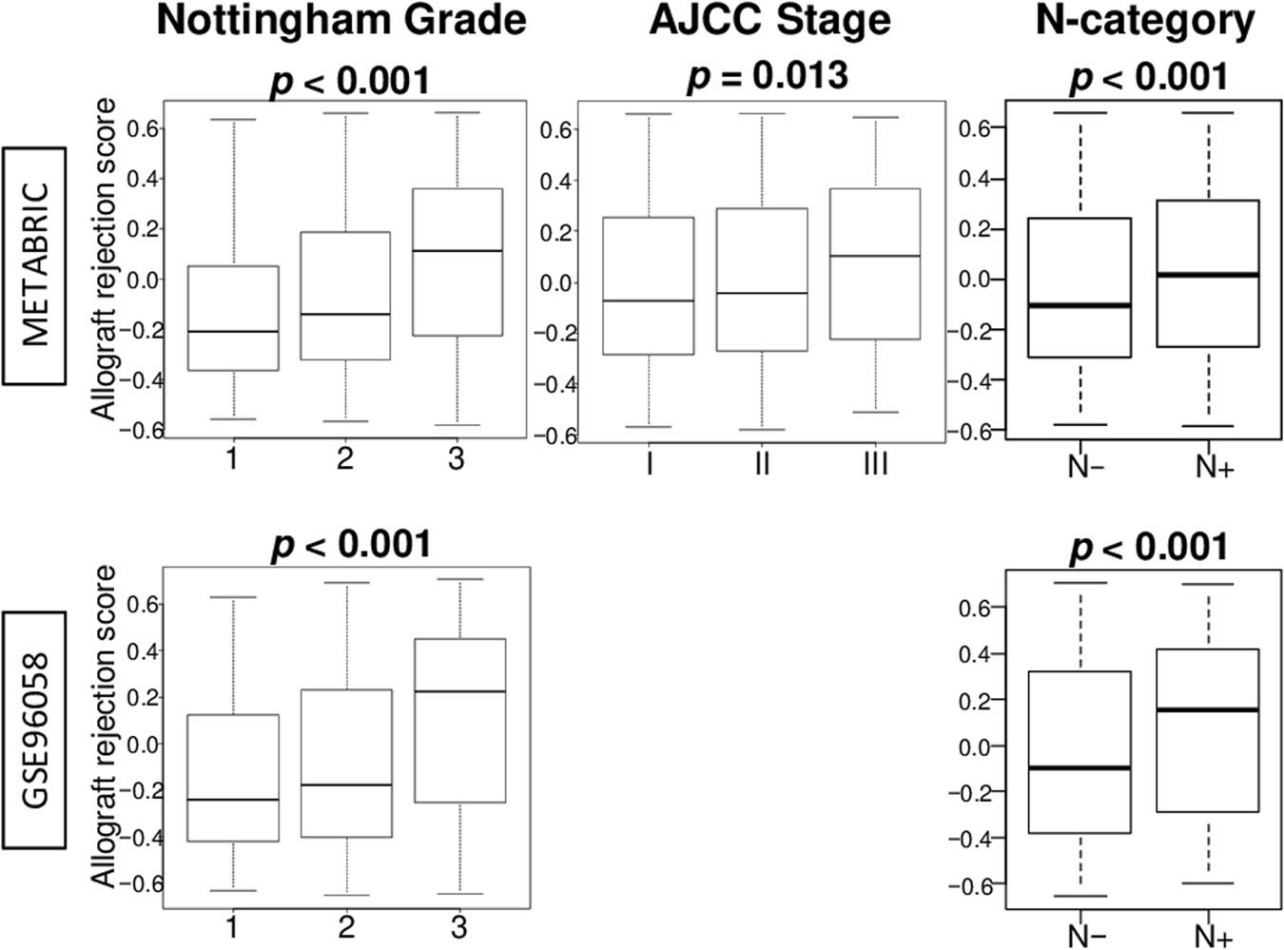
Breast cancer with a high allograft rejection score was significantly associated with aggressive pathological grade, advanced stage, and lymph node metastasis. Boxplots of the score by Nottingham pathological grade, American joint cancer commit (AJCC) stage, and lymph node metastasis status in the METABRIC and GSE96058 cohorts. *p*-values were calculated by Kruskal–Wallis and Mann–Whitney U test. Boxplots were of Tukey type, with boxes depicting median and inter-quartile range.

To investigate the association between the allograft rejection score and cancer aggressiveness, we examined the Nottingham histological grade, American Joint Committee on Cancer (AJCC) staging, and lymph node metastasis status (N-category). We found that the score was significantly associated with advanced grade, advanced stage, and lymph node-positive tumors in the METABRIC cohort (Fig. 4, *p* < 0.001, *p* = 0.013, and *p* < 0.001, respectively). The results of the association of Nottingham histological grade and node-positive status were validated in the GSE96058 cohort (both *p* < 0.001). On the other hand, the score was not associated with any of them within TNBC subtype (Supplementary Fig. 2). This result is in agreement with the notion that the association of the score, grade, and stage is a reflection of the correlation of the score with TNBC, which is known to be associated with a higher grade and stage. We showed the comparison of clinical and pathological features between low and high allograft rejection scores with breast cancer in each cohort (Supplementary Tables 4–6). We also showed the association of other immune-related gene sets with clinical features in the METABRIC cohort in Supplementary Table 7.

### Both immune-related and pro-cancer gene sets were enriched in both triple-negative breast cancer (TNBC) and ER-positive/HER2-negative breast cancer with a high allograft rejection score

**Fig. 5:**
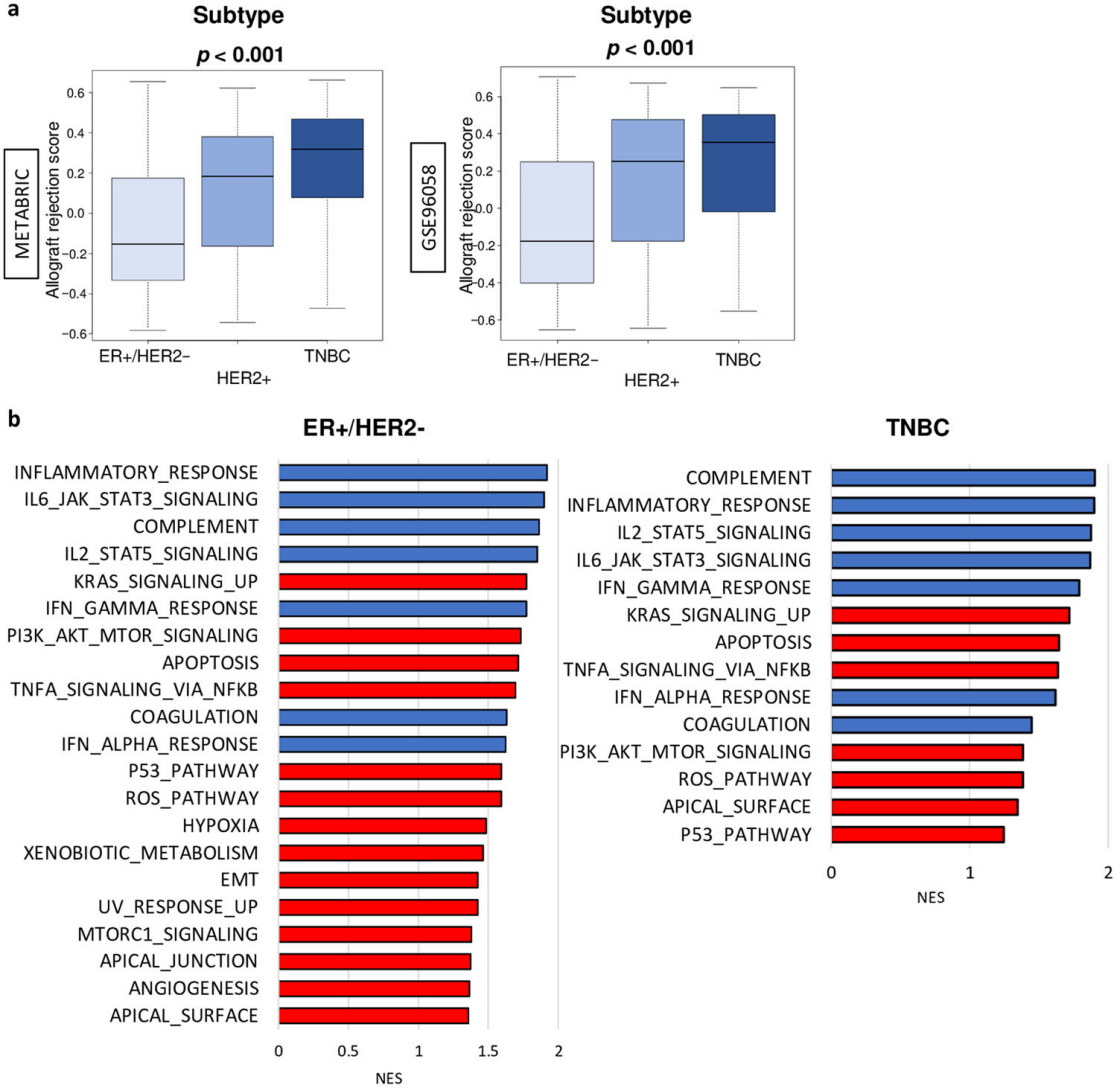
TNBC with a high allograft rejection score was enriched with pro-cancer-related gene sets consistently in the METABRIC and GSE96058 cohorts. **a** Boxplots of the score by breast cancer subtypes in the METABRIC and GSE96058 cohorts. *p*-values were calculated by Kruskal–Wallis. Boxplots were of Tukey type, with boxes depicting median and inter-quartile range. **b** Bar plots of hallmark gene sets, which enriched in high score group within each subtype cohort in the METABRIC cohort. Blue bars are immune-related, and red bars are cancer aggressiveness-related gene sets. Spearman’s rank correlation was used for the analysis. EMT epithelial–mesenchymal transition, IFN interferon ROS reactive oxygen species.

The allograft rejection score levels were compared between the breast cancer subtypes, as there is a known difference in immune cell infiltration. As expected, TNBC was associated with a significantly higher score compared to the other subtypes (Fig. 5a). Of note, the majority of ER-positive/HER2-negative breast cancer had a lower score than the median score of TNBC consistently in both METABRIC and GSE96058 cohorts.

Next, we investigated the underlying mechanism involved in the score by using Gene Set Enrichment Analysis (GSEA) of ER-positive/HER2-negative breast cancer and TNBC. As expected, high score tumors enriched immune-related gene sets: inflammatory response, complement, interferon (IFN)-γ response, IFN-α response, tumor necrosis factor (TNF)-α signaling via NFkB, IL6/JAK/STAT3 signaling, coagulation, p53 pathway, reactive oxygen species (ROS) pathway, and apoptosis in both subtypes in the METABRIC cohort (Fig. 5b and Supplementary Fig. 1). In addition, we found that they also enriched pro-cancer gene sets: KRAS signaling up, PI3K/AKT/MTOR signaling, and apical surface gene sets. Furthermore, high score tumors enriched hypoxia, xenobiotic metabolism, epithelial–mesenchymal transition (EMT), UV response up, apical junction, Mtorc1 response, and angiogenesis in the ER-positive/HER2-negative breast cancer cohort, but not in the TNBC cohort (Fig. 5b). These results were validated by the GSE96058 cohort, which suggests that enhanced immune response is associated with aggressive cancer biology in breast cancer regardless of subtype.

### TNBC with a high allograft rejection score, but not ER-positive/HER2-negative breast cancer, was associated with better survival

**Fig. 6:**
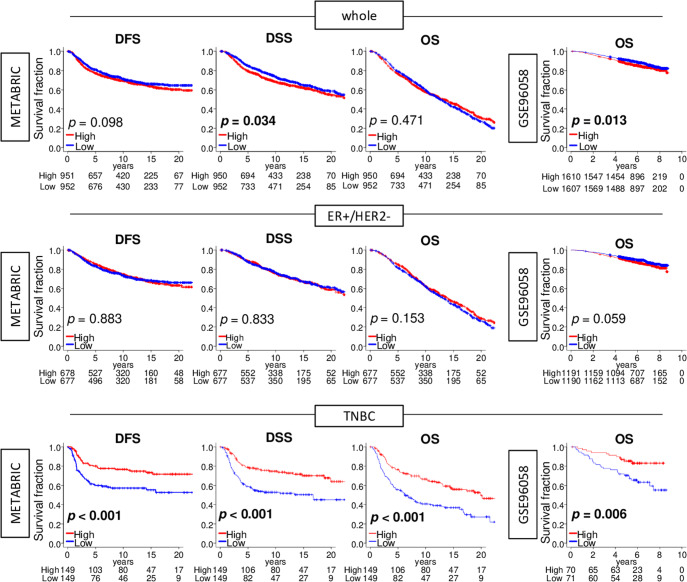
TNBC with a high allograft rejection score is significantly associated with better survival in the METABRIC and GSE96058 cohorts. Kaplan–Meier plots with log-rank *p*-values of OS, DFS, and DSS in the METABRIC cohort, and OS in the GSE96058 cohort between low and high allograft rejection in whole, ER+/HER2−, and TNBC cohorts. *p*-values were calculated by log-rank test. DFS, disease-free survival, DSS disease-specific survival, ER estrogen receptor, HER2 human epidermal growth factor 2, OS overall survival, TNBC triple-negative breast cancer.

Given the significant difference in the allograft rejection score across breast cancer subtypes, we investigated the association of the score with patient survival in whole breast cancer as well as in an estrogen receptor (ER)-positive/human epidermal growth factor 2 (HER2)-negative, and in TNBC in both METABRIC and GSE96058 cohorts. We found whole breast cancer with a high score was significantly associated with worse disease-specific survival (DSS) in the METABRIC cohort and worse overall survival (OS) in the GSE96058 cohort (Fig. 6). There was no survival difference by the score in ER+/HER2− breast cancer patients. On the other hand, TNBC with a high score was significantly associated with better disease-free survival (DFS), DSS, and OS in the METABRIC cohort (all *p* < 0.001) and with OS in the GSE96058 cohort (*p* = 0.006). Furthermore, the score was found to be independently prognostic factor of the other clinical factors in TNBC; Age, AJCC T- and N-category, Nottingham grade, for DFS (hazard ratio (HR) = 2.18, 95% CI = 1.44–3.29, *p* < 0.001), DSS (HR = 2.12, 95% CI = 1.43–3.13, *p* < 0.001), and OS (HR = 1.83, 95% CI = 1.32–2.43, *p* < 0.001) by multivariate cox regression analyses using significant factors by univariate Cox regression analyses, in the METABRIC (Supplementary Table 8). We also found that the allograft rejection score was highest associated with worse TNBC patient survival compared to other immune-related gene sets (Supplementary Table 9). These results suggest that a high allograft score was associated with better survival only in TNBC.

## Discussion

To test our hypothesis that enhanced immune activity measured by computational algorithm is associated with improved patient outcomes similar to previous pathological studies, we analyzed 6245 patients from experimental and validation cohorts using hallmark allograft rejection gene sets as analyzed by the GSVA algorithm. We found that the score correlated with cytolytic activity and all the other hallmark immune-related gene sets. The tumor-infiltrating immune cells, including anti-cancer immune cells (CD4^+^ and CD8^+^ T cells, Th1 and Th2 cells, M1 macrophages, B cells, and pDCs were reflected in the score. The expression levels of immune checkpoint molecular genes were also correlated with the score. A high score was associated with high levels of intratumor heterogeneity, homologous recombination defects, mutation rate, histological grade, advanced stage, and lymph node metastasis. Breast malignancy with a high score enriched immune-related gene sets and pro-cancer-related gene sets, including epithelial–mesenchymal transition and KRAS pathway, in ER-positive/HER2-negative and triple-negative breast cancer (TNBC) groups. TNBC had the highest score compared to other subtypes, and was associated with better survival, whereas that was not the case in the other subtypes. Although it is known that immune cells density is higher in TNBC and is associated with its prognosis, the novelty of this study is that phenomenon was completely echoed using multiple algorithms of in Silico computational biological analyses, which are more economical, objective, and quantifiable compared from classic pathological analyses.

Based on the notion that the accumulation of somatic mutations in cancer cells drives cancer progression^1,37^, we previously reported that aggressive cancer biology and anti-cancer immunity are counterbalanced in breast cancer with high mutation rates^4^. This finding led us to the current study where we investigated the clinical relevance of immune activity in breast cancer. To this end, we utilized the allograft rejection gene set as the score that correlated strongly with cytolytic activity as well as with all the other hallmark immune-related gene sets. We used this score as a measure to quantify immune activity in the tumor microenvironment.

In agreement with our previous study, we found that breast cancer with high immune activity was associated with a high mutation rate and high HRD. However, we did not observe a difference in neoantigens, which suggests that high immune activity by immune cell infiltration may not be due to a high number of neoantigens alone. Nevertheless, breast cancer with high immune activity not only enriched immune-related gene sets, but also enriched pro-cancer gene sets: KRAS signaling, PI3K/AKT/MTOR signaling, and apical surface gene sets. Consistent with these findings, breast cancer with high immune activity was associated with advanced stage, lymph node metastasis, and with advanced histological grade (a commonly used clinical parameter for cancer cell proliferation).

The immune system has been revealed to play a critical role in the initiation and progression of cancers^38^. Interestingly, we found that the level of immune activity was related to survival in only TNBC alone, and not in ER-positive/HER2-negative. Although similar gene sets were enriched in both subtypes in GSEA, and CYT was significantly higher in the high allograft rejection score group in both subtypes (Supplementary Fig. 3), survival only correlated to immune activity TNBC patients. This is in agreement with recent studies that demonstrated that TNBC possesses higher immunogenicity than other breast subtypes^39^. Further, Denkert et al. reported that increased ITLs were associated with better survival in TNBC. However, survival was negatively correlated with immune activity in ER-positive/HER2-negative breast cancer, suggesting a different biology of the immunological infiltrate in this subtype. Although our results are consistent with this is, we cannot help but speculate that the absolute level of immune activity is more clinically relevant than the relative amount within a given cell subtype.

Several studies reveal the association of survival with the existence of tumor-infiltrating lymphocytes (TILs) in TNBC^10,34^, but their function remains unclear. In this study, we demonstrated that the immune response, quantified by the allograft rejection score, is associated with better survival in TNBC, but not in ER-positive/HER2-negative breast cancer. Taken together with our previous finding that a high inflammatory response was significantly associated with better survival in TNBC^29^, it is possible that the immune cell infiltration in TNBC may be due to a higher inflammatory signature exhibited by this subtype. The high immune activity in TNBC with high immune cell infiltration outperform the malignant biology, which is clear from survival outcome. Since the amount of immune cell infiltrations in allograft score high in ER-positive/HER2-negative subtype were roughly same as allograft score low in TNBC, we cannot help but speculate that high allograft score in ER-positive/HER2-negative subtype do not have enough immune cell infiltration to outperform pro-cancer signaling. In addition, some of the pro-cancer signaling such as MTORC1 signaling, angiogenesis, EMT, and hypoxia, were not enriched to high allograft score in TNBC, which suggest that not only enhancement of immune response, but also less malignant biology may contribute to better survival in that subtype. The finding that the allograft rejection score was also not associated with grade or stage/nodal disease in TNBC is somewhat confusing, but at the same time, these are the interesting findings only obtainable when clinical specimens were analyzed. Part of the confusion is that features of biologic aggressiveness, such as enhanced cell proliferation, do not always portend the development of aggressive cancer as measured by advanced stage or metastasis. This is because highly proliferative cells often respond to cytotoxic chemotherapy, thus resulting in better outcomes despite the features of biologic aggressiveness. To the contrary, less proliferative apocrine breast cancer is less likely to respond to chemotherapy, thus resulting in worse clinical outcomes. Previously, we have reported that high mutation breast cancer is associated with highly proliferative cancer, which is counterbalanced by the high infiltration of immune cells and immune response. In agreement, high allograft rejection score cancer that correlates with immune cell infiltration and immune response is associated with cell proliferation as reflected in histological grade. In addition to cell proliferation, the abundance of tumor-infiltrating lymphocytes are known as a surrogate marker of improved drug response and survival. To this end, further studies are warranted to assess the usefulness of a score that quantifies immune activity through immunostaining of tumor-infiltrating lymphocytes or immune checkpoint molecules. There are clinically used gene expression profiles that predict the risk of recurrence in ER-positive/HER2-negative breast cancer, such as Oncotype Dx and Mammaprint. Oncotype Dx utilize 21-genes that represent cell proliferation, invasion, and biomarker receptors. On the other hand, the allograft rejection score is composed of genes selected in the Hallmark collection that are expressed when the rejection of allograft occurs representative of a strong immune reaction. To this end, there is no overlap between genes in the allograft rejection score and these other gene expression profiles. We have previously developed and reported the 3-gene score as a score to predict pCR after chemotherapy in TNBC^40^. It was significantly associated with cell proliferation signaling. The present score was created from a new perspective, and we intend to conduct a prospective study in the future.

There are some limitations to this study. Although our findings were validated by data from a total of 6245 real patients from three completely independent cohorts to minimize the risk of experimental artifact or contamination, this is a retrospective study. These types of studies do not prove any new mechanisms, thus prospective studies and experiments are needed for functional validations. Furthermore, the study was based only on gene expression data from resected breast cancer. The originality of this study stems from our ability to demonstrate the clinical relevance of an immune function score in human breast cancer patients using the largest patient cohorts to date. The current study does not provide nor prove the mechanistic model how enhanced immune response outperform aggressive cancer biology. No experiments were performed to pursue them because our scope was to demonstrate what mechanism translate to the patients in the clinics and we believe no experimental model can duplicate cancer in patients. With that said, prospective studies and experiments are needed for functional validations. The strength of our work is that our results are derived are from the patients’ bulk tumors, which cannot be reliably reproduced in any experimental setting. On the other hand, it is also believed that understanding of the mechanism will be deepened by conducting in vivo and/or in vitro experiments, and the clinical relevance of this score should be confirmed in a prospective study in the future. Finally, analyzed cohorts lack details of specific systemic therapies and it is assumed that all the patients underwent the “standard of care”. This issue is particularly relevant in HER2-positive subtype since anti-HER2 therapy is so effective that whether the patient did or did not receive the therapy is a significant confounder. All cohorts cross the time before and after the use of anti-HER2 therapy, which may explain the differences observed in OS of allograft score high patients in whole cohort of METABRIC and GSE96058. We did exclude the survival analysis of HER2-positive subtype because we did not have access to the information which patient received the anti-HER2 therapy.

In conclusion, we found that breast cancer with a high immune response is associated with aggressive cancer biology—specifically high mutation rate, HRD, intratumoral heterogeneity, advanced histological grade, and stage—using a gene set score that reflects all the gene sets related to immunity, cytolytic activity, and immune cell infiltration. Although tumors with a high immune response were associated with aggressive cancer biology in ER-positive/HER2-negative and triple-negative breast cancer, it was associated with survival only in the latter. Our findings imply that the quantification of immune response using computational biological approach may allow avoidance of cost, labor, and professional bias involved in pathological analyses of immune infiltration within a subtype.

## Methods

### Data acquisition of breast cancer

Clinical information and gene expression data were obtained from 1903 breast cancer patients in the Molecular Taxonomy of Breast Cancer International Consortium (METABRIC) cohort through cBioportal^41,42^. The GSE96058 cohort included data from 3273 breast cancer patients who had a transcriptome profile of resected tumors in an ongoing study. The latest publicly available clinical data for these patients was obtained from the resources listed in the recent study by the Swedish Breast Cancer Analysis Network (SCAN-B)^43^. These two large cohorts were used to test and validate the findings in this study. Transcriptomic data was also obtained on 1069 female breast cancer patients from The Cancer Genome Atlas (TCGA) cohort^44^ for investigating the association of the allograft rejection score with mutation-related score, which were calculated by Thorsson et al.^45^. The GSE75688 cohort has single-cell RNA-sequencing data of tumor cells, stromal cells, immune cells, and myeloid cells in breast cancer^46^, which was obtained from Gene Expression Omnibus (GEO). The approval of the Roswell Park Institutional Review Board was waived due to the deidentified nature of the data points.

### Immune-related gene sets score

Liberzon et al. reported the Molecular Signatures Database (MSigDB) hallmark gene set collection, which is one of the most widely used and comprehensive databases of gene sets for performing gene set enrichment analysis^47^. We used seven gene sets, including allograft rejection, coagulation, complement, interferon (IFN)-α response, IFN-γ response, IL6/JAK/STAT3 signaling, and inflammatory response, which are described immune-related gene sets. Each gene sets score was calculated by Gene Set Variation Analysis (GSVA) algorithm^25^. Of the 200 genes that composed the allograft rejection gene set, METABRIC, GSE96058, and TCGA cohorts contain 183, 199, and 199 genes, respectively.

### Other scores

Cytolytic activity (CYT) score was calculated using two genes, granzyme A (*GZMA*) and perforin (*PRF1*)^11^, which is used as an important marker of tumor inflammation that is indicative of a microenvironment rich in T cells in several research studies^12,36,48,49^. xCell algorithm^16^ was used to estimate the fractions of 64 infiltrating immune cell types as well as stromal cells in each tumor tissue to evaluate intratumor cell composition using multiple gene expression. Mutation-related score, silent and non-silent mutation rate, fraction altered, single nucleotide variation (SNV) and indel neoantigens, homologous recombination defects (HRD), and intratumor heterogeneity were calculated by Thorsson et al.^45^ in the TCGA cohort.

### Gene set enrichment analysis

To explore the difference in signaling pathways enrichment between low- and high-allograft rejection score groups, we performed Gene Set Enrichment Analysis (GSEA)^50^ using GSEA Java software (version 4.1) with MSigDB Hallmark gene sets^47^. False discovery rate (FDR) <25% was used to deem statistical significance, as recommended by GSEA.

### Other statistical analyses

Using R software (version 4.0.1) and Microsoft Excel (version 16), we performed all analyzes and data plots. The analysis of the comparison of groups used the Kruskal–Wallis test, the Mann–Whitney U test, or the Fisher exact test. Survival analysis between two groups was used in the Kaplan–Meier plot with the log-rank test. Values of *p* < 0.05 generally indicate a statistically significant difference.

## Supplementary information

Supplementary information

## Data Availability

All the cohorts/datasets used in this study; Molecular Taxonomy of Breast Cancer International Consortium (METABRIC), GSE96058, The Cancer Genome Atlas (TCGA), and GSE75688, are all publicly available without any restrictions via cBioportal or Gene Expression Omnibus (GEO).
