# Supplementary material for: Enhanced immune response outperform aggressive cancer biology and is associated with better survival in triple-negative breast cancer
Source: NPJ Breast Cancer. 2022 Aug 9;8:92. doi: 10.1038/s41523-022-00466-2 (PMC9363489; doi:10.1038/s41523-022-00466-2)
Supplement: Supplementary file 1 — Supplementary information [file 41523_2022_466_MOESM1_ESM.pdf]

SUPPLEMENTARY INFORMATION

Oshi et al. "Enhanced immune response outperform aggressive cancer biology and is associated with better survival in triple-negative breast cancer"

Contents:

Supplementary Table 1: Gene member in the allograft rejection score.

Supplementary Table 2: Association of immune-related gene sets with infiltration of immune cells.

Supplementary Table 3: The details of the association of allograft rejection with several mutation-related scores in the TCGA cohort.

Supplementary Table 4: Comparison of clinical and pathological features between low and high allograft rejection scores with breast cancer in the METABRIC cohort.

Supplementary Table 5: Comparison of clinical and pathological features between low and high allograft rejection score with breast cancer in the GSE96058 cohort.

Supplementary Table 6: Comparison of clinical and pathological features between low and high allograft rejection score with breast cancer in the TCGA cohort.

Supplementary Table 7: Association of immune-related gene sets with clinical features in the METABRIC cohort.

Supplementary Table 8: Survival analyses of the allograft rejection score and other clinical features of TNBC by uni- and multi-variate analysis in the METABRIC cohort.

Supplementary Table 9: Survival analyses of immune-related gene sets of TNBC by cox regression analysis in the METABRIC cohort.

Supplementary Figure 1: Histogram of allograft rejection score and number of patients (Frequency) in each group of the METABRIC, GSE96058, and TCGA cohorts. Median of each cohort (green line), low (blue arrow) and high (red arrow) groups are shown.

Supplementary Figure 2: Association of the allograft rejection score with Nottingham historogical grade, AJCC stage and lymph node metastasis in TNBC in the METABRIC cohort.

Supplementary Figure 3: Association of the allograft rejection score with cytolytic activity (CYT) in ER-positive/HER2-negative breast cancer.

Supplementary Table 1: Gene member in the allograft rejection score.

Original Member	*Gene Symbol*	*Gene Description*
AARS	*AARS1*	alanyl-tRNA synthetase 1
ABCE1	*ABCE1*	ATP binding cassette subfamily E member 1
ABI1	*ABI1*	abl interactor 1
ACHE	*ACHE*	acetylcholinesterase (Cartwright blood group)
ACVR2A	*ACVR2A*	activin A receptor type 2A
AKT1	*AKT1*	AKT serine/threonine kinase 1
APBB1	*APBB1*	amyloid beta precursor protein binding family B member 1
B2M	*B2M*	beta-2-microglobulin
BCAT1	*BCAT1*	branched chain amino acid transaminase 1
BCL10	*BCL10*	BCL10 immune signaling adaptor
BCL3	*BCL3*	BCL3 transcription coactivator
BRCA1	*BRCA1*	BRCA1 DNA repair associated
C2	*C2*	complement C2
CAPG	*CAPG*	capping actin protein, gelsolin like
CARTPT	*CARTPT*	CART prepropeptide
CCL11	*CCL11*	C-C motif chemokine ligand 11
CCL13	*CCL13*	C-C motif chemokine ligand 13
CCL19	*CCL19*	C-C motif chemokine ligand 19
CCL2	*CCL2*	C-C motif chemokine ligand 2
CCL22	*CCL22*	C-C motif chemokine ligand 22
CCL4	*CCL4*	C-C motif chemokine ligand 4
CCL5	*CCL5*	C-C motif chemokine ligand 5
CCL7	*CCL7*	C-C motif chemokine ligand 7
CCND2	*CCND2*	cyclin D2
CCND3	*CCND3*	cyclin D3
CCR1	*CCR1*	C-C motif chemokine receptor 1
CCR2	*CCR2*	C-C motif chemokine receptor 2
CCR5	*CCR5*	C-C motif chemokine receptor 5
CD1D	*CD1D*	CD1d molecule
CD2	*CD2*	CD2 molecule
CD247	*CD247*	CD247 molecule
CD28	*CD28*	CD28 molecule
CD3D	*CD3D*	CD3d molecule
CD3E	*CD3E*	CD3e molecule
CD3G	*CD3G*	CD3g molecule
CD4	*CD4*	CD4 molecule
CD40	*CD40*	CD40 molecule
CD40LG	*CD40LG*	CD40 ligand
CD47	*CD47*	CD47 molecule
CD7	*CD7*	CD7 molecule
CD74	*CD74*	CD74 molecule
CD79A	*CD79A*	CD79a molecule
CD80	*CD80*	CD80 molecule
CD86	*CD86*	CD86 molecule
CD8A	*CD8A*	CD8a molecule
CD8B	*CD8B*	CD8b molecule
CD96	*CD96*	CD96 molecule
CDKN2A	*CDKN2A*	cyclin dependent kinase inhibitor 2A
CFP	*CFP*	complement factor properdin
CRTAM	*CRTAM*	cytotoxic and regulatory T cell molecule
CSF1	*CSF1*	colony stimulating factor 1
CSK	*CSK*	C-terminal Src kinase
CTSS	*CTSS*	cathepsin S
CXCL13	*CXCL13*	C-X-C motif chemokine ligand 13

CXCL9	*CXCL9*	C-X-C motif chemokine ligand 9
CXCR3	*CXCR3*	C-X-C motif chemokine receptor 3
DARS	*DARS1*	aspartyl-tRNA synthetase 1
DEGS1	*DEGS1*	delta 4-desaturase, sphingolipid 1
DYRK3	*DYRK3*	dual specificity tyrosine phosphorylation regulated kinase 3
EGFR	*EGFR*	epidermal growth factor receptor
EIF3A	*EIF3A*	eukaryotic translation initiation factor 3 subunit A
EIF3D	*EIF3D*	eukaryotic translation initiation factor 3 subunit D
EIF3J	*EIF3J*	eukaryotic translation initiation factor 3 subunit J
EIF4G3	*EIF4G3*	eukaryotic translation initiation factor 4 gamma 3
EIF5A	*EIF5A*	eukaryotic translation initiation factor 5A
ELANE	*ELANE*	elastase, neutrophil expressed
ELF4	*ELF4*	E74 like ETS transcription factor 4
EREG	*EREG*	epiregulin
ETS1	*ETS1*	ETS proto-oncogene 1, transcription factor
F2	*F2*	coagulation factor II, thrombin
F2R	*F2R*	coagulation factor II thrombin receptor
FAS	*FAS*	Fas cell surface death receptor
FASLG	*FASLG*	Fas ligand
FCGR2B	*FCGR2B*	Fc fragment of IgG receptor Iib
FGR	*FGR*	FGR proto-oncogene, Src family tyrosine kinase
FLNA	*FLNA*	filamin A
FYB	*FYB1*	FYN binding protein 1
GALNT1	*GALNT1*	polypeptide N-acetylgalactosaminyltransferase 1
GBP2	*GBP2*	guanylate binding protein 2
GCNT1	*GCNT1*	glucosaminyl (N-acetyl) transferase 1
GLMN	*GLMN*	glomulin, FKBP associated protein
GPR65	*GPR65*	G protein-coupled receptor 65
GZMA	*GZMA*	granzyme A
GZMB	*GZMB*	granzyme B
HCLS1	*HCLS1*	hematopoietic cell-specific Lyn substrate 1
HDAC9	*HDAC9*	histone deacetylase 9
HIF1A	*HIF1A*	hypoxia inducible factor 1 subunit alpha
HLA-A	*HLA-A*	major histocompatibility complex, class I, A
HLA-DMA	*HLA-DMA*	major histocompatibility complex, class II, DM alpha
HLA-DMB	*HLA-DMB*	major histocompatibility complex, class II, DM beta
HLA-DOA	*HLA-DOA*	major histocompatibility complex, class II, DO alpha
HLA-DOB	*HLA-DOB*	major histocompatibility complex, class II, DO beta
HLA-DQA1	*HLA-DQA1*	major histocompatibility complex, class II, DQ alpha 1
HLA-DRA	*HLA-DRA*	major histocompatibility complex, class II, DR alpha
HLA-E	*HLA-E*	major histocompatibility complex, class I, E
HLA-G	*HLA-G*	major histocompatibility complex, class I, G
ICAM1	*ICAM1*	intercellular adhesion molecule 1
ICOSLG	*ICOSLG*	inducible T cell costimulator ligand
IFNAR2	*IFNAR2*	interferon alpha and beta receptor subunit 2
IFNG	*IFNG*	interferon gamma
IFNGR1	*IFNGR1*	interferon gamma receptor 1
IFNGR2	*IFNGR2*	interferon gamma receptor 2
IGSF6	*IGSF6*	immunoglobulin superfamily member 6
IKBKB	*IKBKB*	inhibitor of nuclear factor kappa B kinase subunit beta
IL10	*IL10*	interleukin 10
IL11	*IL11*	interleukin 11
IL12A	*IL12A*	interleukin 12A
IL12B	*IL12B*	interleukin 12B
IL12RB1	*IL12RB1*	interleukin 12 receptor subunit beta 1
IL13	*IL13*	interleukin 13

IL15	*IL15*	interleukin 15
IL16	*IL16*	interleukin 16
IL18	*IL18*	interleukin 18
IL18RAP	*IL18RAP*	interleukin 18 receptor accessory protein
IL1B	*IL1B*	interleukin 1 beta
IL2	*IL2*	interleukin 2
IL27RA	*IL27RA*	interleukin 27 receptor subunit alpha
IL2RA	*IL2RA*	interleukin 2 receptor subunit alpha
IL2RB	*IL2RB*	interleukin 2 receptor subunit beta
IL2RG	*IL2RG*	interleukin 2 receptor subunit gamma
IL4	*IL4*	interleukin 4
IL4R	*IL4R*	interleukin 4 receptor
IL6	*IL6*	interleukin 6
IL7	*IL7*	interleukin 7
IL9	*IL9*	interleukin 9
INHBA	*INHBA*	inhibin subunit beta A
INHBB	*INHBB*	inhibin subunit beta B
IRF4	*IRF4*	interferon regulatory factor 4
IRF7	*IRF7*	interferon regulatory factor 7
IRF8	*IRF8*	interferon regulatory factor 8
ITGAL	*ITGAL*	integrin subunit alpha L
ITGB2	*ITGB2*	integrin subunit beta 2
ITK	*ITK*	IL2 inducible T cell kinase
JAK2	*JAK2*	Janus kinase 2
KLRD1	*KLRD1*	killer cell lectin like receptor D1
KRT1	*KRT1*	keratin 1
LCK	*LCK*	LCK proto-oncogene, Src family tyrosine kinase
LCP2	*LCP2*	lymphocyte cytosolic protein 2
LIF	*LIF*	LIF interleukin 6 family cytokine
LTB	*LTB*	lymphotoxin beta
LY75	*LY75*	lymphocyte antigen 75
LY86	*LY86*	lymphocyte antigen 86
LYN	*LYN*	LYN proto-oncogene, Src family tyrosine kinase
MAP3K7	*MAP3K7*	mitogen-activated protein kinase kinase kinase 7
MAP4K1	*MAP4K1*	mitogen-activated protein kinase kinase kinase kinase 1
MBL2	*MBL2*	mannose binding lectin 2
MMP9	*MMP9*	matrix metallopeptidase 9
MRPL3	*MRPL3*	mitochondrial ribosomal protein L3
MTIF2	*MTIF2*	mitochondrial translational initiation factor 2
NCF4	*NCF4*	neutrophil cytosolic factor 4
NCK1	*NCK1*	NCK adaptor protein 1
NCR1	*NCR1*	natural cytotoxicity triggering receptor 1
NLRP3	*NLRP3*	NLR family pyrin domain containing 3
NME1	*NME1*	NME/NM23 nucleoside diphosphate kinase 1
NOS2	*NOS2*	nitric oxide synthase 2
NPM1	*NPM1*	nucleophosmin 1
PF4	*PF4*	platelet factor 4
PRF1	*PRF1*	perforin 1
PRKCB	*PRKCB*	protein kinase C beta
PRKCG	*PRKCG*	protein kinase C gamma
PSMB10	*PSMB10*	proteasome 20S subunit beta 10
PTPN6	*PTPN6*	protein tyrosine phosphatase non-receptor type 6
PTPRC	*PTPRC*	protein tyrosine phosphatase receptor type C
RARS	*RARS1*	arginyl-tRNA synthetase 1
RIPK2	*RIPK2*	receptor interacting serine/threonine kinase 2
RPL39	*RPL39*	ribosomal protein L39

RPL3L	*RPL3L*	ribosomal protein L3 like
RPL9	*RPL9*	ribosomal protein L9
RPS19	*RPS19*	ribosomal protein S19
RPS3A	*RPS3A*	ribosomal protein S3A
RPS9	*RPS9*	ribosomal protein S9
SIT1	*SIT1*	signaling threshold regulating transmembrane adaptor 1
SOCS1	*SOCS1*	suppressor of cytokine signaling 1
SOCS5	*SOCS5*	suppressor of cytokine signaling 5
SPI1	*SPI1*	Spi-1 proto-oncogene
SRGN	*SRGN*	serglycin
ST8SIA4	*ST8SIA4*	ST8 alpha-N-acetyl-neuraminide alpha-2,8-sialyltransferase 4
STAB1	*STAB1*	stabilin 1
STAT1	*STAT1*	signal transducer and activator of transcription 1
STAT4	*STAT4*	signal transducer and activator of transcription 4
TAP1	*TAP1*	transporter 1, ATP binding cassette subfamily B member
TAP2	*TAP2*	transporter 2, ATP binding cassette subfamily B member
TAPBP	*TAPBP*	TAP binding protein
TGFB1	*TGFB1*	transforming growth factor beta 1
TGFB2	*TGFB2*	transforming growth factor beta 2
THY1	*THY1*	Thy-1 cell surface antigen
TIMP1	*TIMP1*	TIMP metallopeptidase inhibitor 1
TLR1	*TLR1*	toll like receptor 1
TLR2	*TLR2*	toll like receptor 2
TLR3	*TLR3*	toll like receptor 3
TLR6	*TLR6*	toll like receptor 6
TNF	*TNF*	tumor necrosis factor
TPD52	*TPD52*	tumor protein D52
TRAF2	*TRAF2*	TNF receptor associated factor 2
TRAT1	*TRAT1*	T cell receptor associated transmembrane adaptor 1
UBE2D1	*UBE2D1*	ubiquitin conjugating enzyme E2 D1
UBE2N	*UBE2N*	ubiquitin conjugating enzyme E2 N
WARS	*WARS1*	tryptophanyl-tRNA synthetase 1
WAS	*WAS*	WASP actin nucleation promoting factor
ZAP70	*ZAP70*	zeta chain of T cell receptor associated protein kinase 70

Supplementary Table 2: Association of immune-related gene sets with infiltration of immune cells.

Immune-related gene sets	$CD8^+$ T cells	$CD4^+$ memory T cells	Th1 cells	M1 macrophages	pDC	NK cells	Tregs	Th2 cells	M2 macrophages	B cells
Coagulation	0.213	0.26	-0.498	0.103	0.31	0.492	0.011	0.207	0.211	0.258
Complement	0.519	0.725	-0.168	0.607	0.73	0.542	0.385	0.564	0.271	0.586
IFNα response	0.486	0.564	0.174	0.689	0.629	0.492	0.355	0.372	0.137	0.524
IFNγ response	0.608	0.706	0.079	0.733	0.753	0.591	0.385	0.509	0.177	0.64
IL6/JAK/STAT3 signaling	0.623	0.716	-0.122	0.612	0.747	0.559	0.342	0.512	0.184	0.644
Inflammatory response	0.547	0.685	-0.119	0.627	0.722	0.541	0.35	0.537	0.236	0.258
Allograft rejection	0.749	0.832	-0.035	0.711	0.839	0.668	0.378	0.582	0.206	0.74

Supplementary Table 3: The details of the association of allograft rejection with several mutation-related scores in the TCGA cohort.

Score		Low	High
Silent mutation rate	Range	0.00-36.18	0.00-35.13
	IQR	0.16-0.47	0.18-0.64
	Median	0.28	0.35
Non-silent mutation rate	Range	0.00-113.65	0.00-151.47
	IQR	0.56-1.36	0.61-2.09
	Median	0.82	1.05
Fraction altered	Range	0.00-1.00	0.00-0.99
	IQR	0.16-0.74	0.20-0.67
	Median	0.43	0.45
SNV neoantigens	Range	2-1838	0-1995
	IQR	10-27	12-41
	Median	16	22
Indel neoantigens	Range	0-990	0-1578
	IQR	1-36	2-44
	Median	7	10
HRD	Range	0-89	0-85
	IQR	9-30	11-42
	Median	17	23
Intratumor heterogeneity	Range	0.00-0.96	0.00-0.84
	IQR	0.02-0.18	0.04-0.26
	Median	0.07	0.11

HRD, homologous recombination deficiency; IQR, interquartile range; SNV, single nucleotide variants.

Supplementary Table 4. Comparison of clinical and pathological features between low and high allograft rejection scores with breast cancer in the METABRIC cohort.

METABRIC Characteristics		Low (*n* = 952)	High (*n* = 952)	*p*-value
Age	Median	64	60	< 0.001
	IQR	54-72	50-69	
Subtype	ER+/HER2-	819	536	< 0.001
	TNBC	53	245	
	HER2+	78	158	
	Unknown	2	13	
AJCC				
N-category	N-	539	453	<0.001
	N+	413	498	
	Unknown	0	1	
Stage	0	2	2	0.033
	1	251	224	
	2	397	403	
	3	43	72	
	4	6	3	
	Unknown	253	248	
Nottingham Grade	1	115	50	< 0.001
	2	432	308	
	3	365	563	
	Unknown	40	31	

AJCC, American Joint Committee of Cancer; ER, estrogen receptor; HER2, human epidermal growth factor receptor 2; IQR, interquartile range; TNBC, triple negative breast cancer

Supplementary Table 5. Comparison of clinical and pathological features between low and high allograft rejection scores with breast cancer in the GSE96058 cohort.

GSE96058 Characteristics		Low (n = 1636)	High (n = 1637)	p-value
Age	Median	65	63	< 0.001
	IQR	53-72	52-71	
Subtype	ER+/HER2-	1379	1046	< 0.001
	TNBC	34	109	
	HER2+	133	287	
	Unknown	90	195	
AJCC				
T-category	T0	202	214	0.690
	T1	869	827	
	T2	487	513	
	T3	33	38	
	T4	8	7	
	Unknown	41	38	
N-category	N-	1417	1327	< 0.001
	N+	182	275	
	Unknown	37	35	
M-category	M-	1606	1608	1.00
	M+	1	2	
	Unknown	29	27	
Nottingham Grade	1	317	179	< 0.001
	2	886	646	
	3	415	769	
	Unknown	18	43	

AJCC, American Joint Committee of Cancer; ER, estrogen receptor; HER2, human epidermal growth factor receptor 2; IQR, interquartile range; TNBC, triple negative breast cancer

Supplementary Table 6. Comparison of clinical and pathological features between low and high allograft rejection scores with breast cancer in the TCGA cohort.

TCGA Characteristics		Low (*n* = 534)	High (*n* = 535)	*p*-value
Age	Median			
	IQR			
Subtype	ER+/HER2-	324	257	< 0.001
	TNBC	36	123	
	HER2+	84	92	
	Unknown	90	63	
AJCC				
Stage	I	91	87	0.270
	II	295	309	
	III	121	122	
	IV	13	5	
	Unknown	14	12	
T-category	T1	128	145	0.617
	T2	300	316	
	T3	77	60	
	T4	25	13	
	Unknown	2	3	
N-category	N-	253	253	0.665
	N+	263	278	
	Unknown	18	4	
M-category	M-	439	448	0.075
	M+	14	6	
	Unknown	81	81	

AJCC, American Joint Committee of Cancer; ER, estrogen receptor; HER2, human epidermal growth factor receptor 2; IQR, interquartile range; TNBC, triple negative breast cancer

Supplementary Table 7: Association of immune-related gene sets with clinical features in the METABRIC cohort.

	Grade	Stage	N-category	Subtype
Coagulation	<0.001*	0.053	0.388	0.355
Complement	<0.001	0.021	<0.001	<0.001
IFNα response	<0.001	0.002	<0.001	<0.001
IFNγ response	<0.001	<0.001	<0.001	<0.001
IL6/JAK/STAT3 signaling	<0.001	0.11	<0.001	<0.001
Inflammatory response	<0.001	0.023	<0.001	<0.001
Allograft rejection	<0.001	0.013	<0.001	<0.001

Supplementary Table 8: Survival analyses of the allograft rejection score and other clinical features of TNBC by uni- and multi-variate analysis in the METABRIC cohort.

	OS	Univariate				Multivariate			
Clinical factor		HR	95%CI	*p*		HR	95%CI	*p*	
Age	High vs. Low	1.02	1.01-1.04	<0.001	*	1.03	1.02-1.04	<0.001	*
T	T3/4 vs. T1/2	1.64	1.18-2.28	0.003	*	1.34	0.95-1.89	0.091	*
N	N+ vs. N-	1.42	1.03-1.94	0.030	*	1.73	1.23-2.42	0.001	*
Stage	III/IV vs. I/II	2.18	1.33-3.57	0.002	*	-			
Grade	G3 vs. G1/2	1.01	0.65-1.57	0.962		-			
Allograft	Low vs. High	1.93	1.41-2.65	<0.001	*	1.83	1.32-2.43	<0.001	*
	DFS	Univariate				Multivariate			
Clinical factor		HR	95%CI	p		HR	95%CI	p	
Age	High vs. Low	0.99	0.98-1.01	0.282					
T	T3/4 vs. T1/2	1.51	0.99-2.29	0.054					
N	N+ vs. N-	1.95	1.29-2.95	0.002	*	2.05	1.36-3.11	<0.001	*
Stage	III/IV vs. I/II	2.46	1.32-4.59	0.005	*				
Grade	G3 vs. G1/2	1.35	0.72-2.53	0.347					
Allograft	Low vs. High	2.08	1.38-3.14	<0.001	*	2.18	1.44-3.29	<0.001	*
	DSS	Univariate				Multivariate			
Clinical factor		HR	95%CI	p		HR	95%CI	p	
Age	High vs. Low	0.99	0.98-1.01	0.469					
T	T3/4 vs. T1/2	1.65	1.11-2.45	0.013	*	1.3	0.87-1.95	0.203	
N	N+ vs. N-	2.09	1.42-3.09	<0.001	*	2.06	1.38-3.08	<0.001	*
Stage	III/IV vs. I/II	2.87	1.68-4.91	<0.001	*				
Grade	G3 vs. G1/2	1.2	0.68-2.09	0.530					
Allograft	Low vs. High	2.15	1.46-3.16	<0.001	*	2.12	1.43-3.13	<0.001	*

Supplementary Table 9: Survival analyses of immune-related gene sets of TNBC by cox regression analysis in the METABRIC cohort.

TNBC	DFS				DSS				OS			
(median)	HR	95%CI	*p*		HR	95%CI	*p*		HR	95%CI	*p*	
Coagulation	1.06	0.73-1.55	0.748		1.1	0.76-1.59	0.620		1.12	0.82-1.53	0.483	
Complement	1.3	0.89-1.90	0.181		1.4	0.96-2.04	0.076		1.26	0.92-1.73	0.144	
IFNα response	1.16	0.80-1.70	0.432		1.23	0.85-1.78	0.28		1.33	0.97-1.81	0.078	
IFNγ response	1.45	0.99-2.13	0.056		1.46	1.00-2.12	0.048	*	1.47	1.08-2.02	0.016	*
IL6/JAK/STAT3 signaling	1.58	1.08-2.32	0.02	*	1.63	1.12-2.37	0.011	*	1.63	1.19-2.23	0.002	*
Inflammatory response	1.43	0.98-2.10	0.064		1.59	1.09-2.32	0.015	*	1.48	1.08-2.02	0.015	*
Allograft rejection	1.87	1.27-2.76	<0.001	*	2.15	1.46-3.16	<0.001	*	1.93	1.41-2.65	<0.001	*

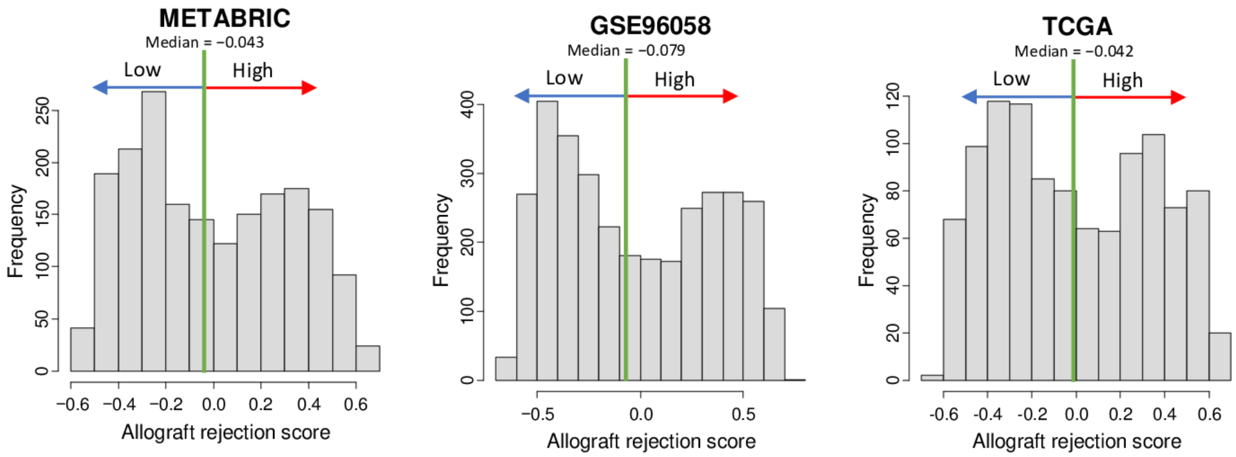

Supplementary Figure 1. Histogram of allograft rejection score and number of patients (Frequency) in each group of the METABRIC, GSE96058, and TCGA cohorts. Median of each cohort (green line), low (blue arrow) and high (red arrow) groups are shown.

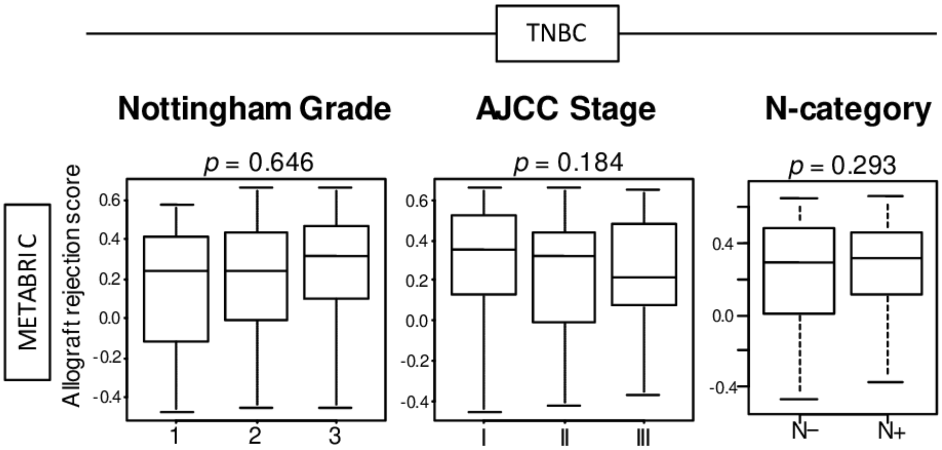

Supplementary Figure 2. Association of the allograft rejection score with Nottingham historogical grade, AJCC stage and lymph node metastasis in TNBC in the METABRIC cohort. Box plots of the score by Nottingham pathological grade and AJCC stage in TNBC subgroup. *p*-values were calculated by Kruskal-Wallis and Mann-Whitney U test.

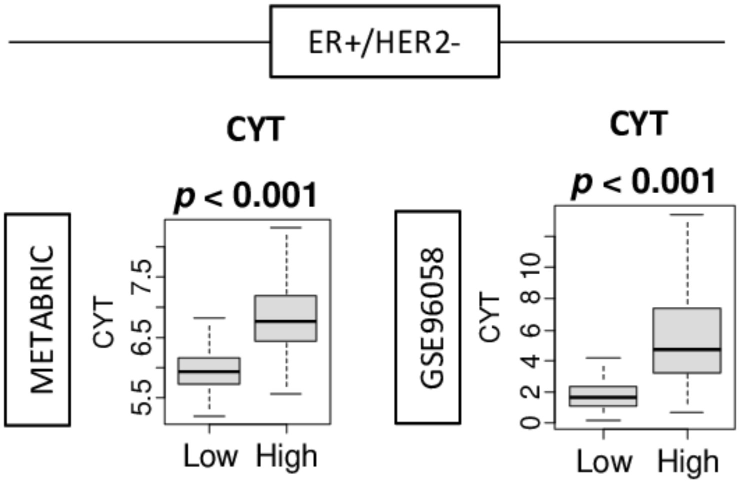

Supplementary Figure 3. Association of the allograft rejection score with cytolytic activity (CYT) in ER-positive/HER2-negative breast cancer. Boxplots of CYT score separated by low and high allograft rejection scores in ER-positive/HER2-negative breast cancer in the METABRIC and GSE96058 cohorts. The median value cut-off was used to divide the two groups within each cohort. *p*-values were calculated by Mann-Whitney U test.
